# Patterns of Physical Activity and Self-rated Health Among Adult Populations in South Asia

**DOI:** 10.5195/cajgh.2020.347

**Published:** 2020-03-31

**Authors:** Sanni Yaya, Ghose Bishwajit

**Affiliations:** 1 Faculty of Social Sciences, School of International Development and Global Studies, University of Ottawa, Canada; 2 Institute of Nutrition and Food science, University of Dhaka, Bangladesh

**Keywords:** Moderate Physical Activity, Vigorous Physical Activity, Self-Rated Health, South Asia, World Health Survey

## Abstract

**Introduction:**

Although South Asians are considered to be at high risk for cardiovascular diseases, research evidence on the health impacts of physical activity (PA) remains very limited. In this study we aimed to explore the patterns of PA and to investigate whether engaging in regular PA is associated with better Self-Rated Health (SRH) among South Asians.

**Methods:**

Cross-sectional data on population health were drawn from the World Health Survey of WHO. Subjects were 28,020 male and female South Asians (from Bangladesh, India, Nepal, and Sri Lanka) aged 18 years and above. Data were analysed using descriptive and multivariable logistic regression analyses.

**Results:**

The proportion of the sample population reported good SRH was 44.3%, 58.7%, 37.7%, and 73.7% in Bangladeshis, Indians, Nepalese, and Sri Lankans, respectively. Regular engagement in moderate PA was highest in Nepal (69.7%) and lowest in Bangladesh (37.4%). Vigorous PA was highest in India (29.9%) and lowest in Bangladesh (17.9%). In Bangladesh, compared to those never engaged in MPA, those who engaged for 1-2, 3-4, 5-6, or 7 days a week were 30% [AOR=1.306; 95%CI 1.085-1.572], 33% [AOR=1.326; 95%CI 1.093-1.609], 39% [AOR=1.389; 95%CI 1.125-1.716], and 46% [AOR=1.459; 95%CI 1.249-1.705] more likely to report being in good health, respectively.

**Conclusions:**

We found that self-reported engagement in physical activities varies in South Asian countries. Since engaging in PA may help improve subjective and objective health status, health policy makers need to focus on designing exercise-friendly neighbourhoods in an attempt to promote population health.

## Introduction

The construct of self-rated health (SRH) is an inclusive measure of public health, and it is used as a reliable predictor of quality of life, subjective well-being, disability, morbidity, and mortality^1^^,^^2^. SRH is one of the most widely used predictors of health risk and prognosis compared with/to other objective measures^3^. Possible explanations for the efficacy of self-assessments of health include its multifaceted representation of an individual's general perception of health, including biological, psychosocial, and cultural dimensions of health and expressiveness^4^. SRH has also been found to be related to clinical measures of health^3^, and it was proposed that general practitioners can utilize SRH measures in clinical encounters^5^. Moreover, SRH can be regarded as a more inclusive measure of health status than clinical diagnosis, as it tends to be sensitive to social determinants of health such as education, socioeconomic status, and living conditions, which have direct influences on health and well-being and on shaping individuals perception of health and illness^4^^,^^5^. SRH is therefore able to provide information above and beyond typical clinical evaluation and thus offers a comprehensive way of assessing a patients' overall health status^4^^,^^6^.

In recent years, there has been an increasing research interest on the impact of various lifestyle factors and health related behaviours on SRH^7^^,^^8^. Behavioural aspects, such as tobacco smoking, alcohol drinking, dietary habits, and engaging in physical activity (PA) are explored in relation to how they correlate with SRH among people of different age groups and socioeconomic backgrounds. Physical inactivity is regarded as a growing public health issue both in developed and developing countries. In Europe and other industrialised societies, increasing sedentary lifestyle has been shown to be associated with worse health and all-cause mortality, independent of level of PA^9^^–^^11^. According to WHO, public health burden of physical inactivity is high and causes an estimated 600,000 deaths per year in Europe alone^11^. Worldwide, physical inactivity is responsible for 6% of the burden of coronary heart disease, 7% of type 2 diabetes, and 10% of breast and colon cancer^12^. It has also been identified as the fourth leading risk factor for global mortality in 2010, accounting for roughly 13.4 million disability adjusted life years (DALYs)^15^ and 6% of all deaths^13^.

There is a growing consensus that moderate- to vigorous-intensity PA has a key preventive role in non-communicable diseases (NCDs), including obesity, cardiovascular disease, type-2 diabetes, and some cancers^9^^,^^10^. Lack of PA during adolescence was reported to be a significant predictor of abdominal obesity in young adulthood leading to a self-perpetuating vicious circle of obesity and physical inactivity^14^. In addition to its contribution to increased morbidity and mortality, physical inactivity is also responsible for a substantial economic burden. Epidemiological evidence on the role of PA on SRH is necessary for making informed health policies that can promote PA in the general population.

South Asians are people who identify with the cultures of Bangladesh, India, Sri Lanka, and Nepal, and account for about a quarter of the global population with a unique epidemiological and sociodemographic profile. Though South Asians are considered at-risk population for cardiovascular diseases, research evidence on the health impacts of PA in this population remains very limited. Therefore, epidemiological evidence from other regions may not be applicable for the population in this region. This study was carried out to provide insights on the pattern of PA in South Asians, and to investigate the association between frequency of PA and SRH among the adult population. Data used in this study were extracted from the World Health Survey program of WHO conducted during 2002-2004 that included four South Asian countries: Bangladesh, India, Nepal, and Sri Lanka.

## Methods

### Data source

This study was based on the data extracted from WHO World Health Survey conducted between 2002 and 2004, available from WHO upon request. Objectives of the WHO-funded survey were to provide reliable and nationally comparable data on a wide range of health and socioeconomic indicators to facilitate evidence-based health policy making. These data are utilized by many researchers due to lack of more recent data on health behaviour and self-rated health in South Asian population. The program is operational in 70 countries including four South Asian countries: Bangladesh, India, Nepal, and Sri Lanka. Further details regarding the original survey study are published elsewhere^16^.

### Variables of interest

Self-rated health status was the outcome variable in this study. Emerging evidence suggests predictability of SRH for both non-clinical and clinical outcomes, and it is being proposed to family physicians as an efficient yet simple way for therapeutic decision making^28^^,^^29^. Respondents were asked to rate their health on a scale from 1 to 5 with the following response options: 1. Very good; 2. Good; 3. Moderate; 4. Bad; and 5. Very Bad. SRH was analysed dichotomously as: 1) Good SRH (Very good and Good), and 2) Poor SRH (Moderate, Bad and Very Bad)^30^. The validity of the single-item tool to measure subjective health was mentioned in previous studies^31^^,^^32^.

The predictor variable of primary interest was PA. The two types of PA used in this study were moderate PA (MPA) and vigorous PA (VPA). VPA was measured by the following question: “Vigorous activities make you breathe much harder than normal and may include heavy lifting, digging, aerobics, or fast bicycling. Think only about those physical activities that you performed for at least 10 minutes at a time. During the last 7 days, on how many days did you do vigorous physical activities?” MPA was measured by the following question: “Moderate physical activities make you breathe somewhat harder than normal and may include carrying light loads, bicycling at a regular pace, or doubles tennis. Do not include walking. Again, think about only those physical activities that you performed for at least 10 minutes at a time. During the last 7 days, on how many days did you do moderate physical activities?”

Answers ranged from 0 to 7 days and were categorised as follows: 0 days (never), 1-2 days, 3-4 days, 5-6 days, and every day.

The other potential predictor variables included in the study were: Age (18-29/30-39/40-49/50-59/60+ years); Sex (Female/Male); Currently married (No/Yes); Educational attainment (Nil/Less than primary school/Primary complete/Secondary complete/High school/equivalent complete/Pre-university/University); Employment status (Government employee/Private employee/Employer/Unemployed); Smoking habit (Daily/Yes, but not daily/Non-smoker); Ever drank alcohol (Yes/No).

### Ethical considerations

Informed Consent was obtained from the recruited participants before their participation in the survey. Participation was completely voluntary, and the respondent had the choice to refuse to take part in the interview. The data used in this study were secondary, which are available in the public domain in anonymised form. Additional approval was therefore was not necessary according to WHO regulations (https://www.who.int/healthinfo/survey/en/).

### Statistical analysis

Datasets were checked for missing values and outliers. Data were cleaned to retain the maximum number of observations. Sample characteristics were analysed through univariate analysis. Cross tabulation was used to measure the distribution of the sociodemographic variables across the outcome SRH variable. Chi-square tests were conducted to assess the group differences for Good vs. Poor self-rated health. Variables that had a p-value below 0.25 were entered into the final regression analysis^17^. Four separate regression models were run for each country. The outcomes of the regression (binary logistic) analyses were reported in terms of adjusted odds ratios (AOR) and corresponding 95% confidence intervals. All analyses were performed with SPSS version 22.

## Results

Descriptive sample characteristics were provided in Table 1. In short, the mean age was highest in Sri Lankans (40.78, SD 15.22) and lowest in Bangladeshis (38.47, SD 14.81). The majority of the participants were between 18 and 29 years of age, female, and currently married. Rate of literacy was highest for Sri Lanka (94.4%) and lowest in Nepal (40%). However, the rate of pre-university/university level education was highest in India (10.1%), followed by Bangladesh (4.3%) and Sri Lanka (2.4%). Regular engagement in MPA was highest for Nepal (69.7%), followed by India (57.6%), Sri Lanka (49.7%), and Bangladesh (37.4%), and that for VPA was highest in India (29.9%) followed by Nepal (24.4%), Sri Lanka (24.1%), and Bangladesh (17.9%).

**Table 1.** Sample characteristics

Variables	Bangladesh		India		Nepal		Sri Lanka	
	(n=5462)		(n=8853)		(n=8031)		(n=5674)	
**Age, Mean (SD)**	38.47 (14.81)		38.69 (15.07)		38.55 (15.33)		40.78 (15.22)	
18–29	1688	30.9	2771	31.3	2666	33.2	1515	26.7
30–39	1502	27.5	2275	25.7	2008	25	1367	24.1
40–49	1103	20.2	1611	18.2	1462	18.2	1231	21.7
50–59	574	10.5	1054	11.9	867	10.8	823	14.5
60+	595	10.9	1151	13	1028	12.8	743	13.1
**Sex**								
Female	2917	53.4	4515	51	4602	57.3	2968	52.3
Male	2545	46.6	4338	49	3429	42.7	2706	47.7
**Currently married**								
No	1218	22.3	2036	23	1413	17.6	1765	31.1
Yes	4244	77.7	6817	77	6618	82.4	3909	68.9
**Educational attainment**								
Nil	2245	41.1	3400	38.4	4819	60	318	5.6
Less than primaiy school	1000	18.3	832	9.4	883	11	431	7.6
Primary complete	1360	24.9	44	0.5	1108	13.8	1430	25.2
Secondary complete	404	7.4	1567	17.7	819	10.2	2236	39.4
High school/equivalent complete	218	4	1142	12.9	257	3.2	1123	19.8
Pre-university/University	235	4.3	894	10.1	137	1.7	136	2.4
**Employment status**								
Govt, employee	197	3.6	336	3.8	249	3.1	511	9
Private employee	333	6.1	974	11	169	2.1	647	11.4
Employer	2081	38.1	3621	40.9	5381	67	1923	33.9
Not working for payment	2851	52.2	3940	44.5	2225	27.7	2599	45.8
**Smoking habit**								
Daily	2021	37	2780	31.4	3212	40	726	12.8
Yes. not daily	300	5.5	266	3	385	4.8	460	8.1
Non-smoker	3141	57.5	5808	65.6	4433	55.2	4494	79.2
Ever drank alcohol								
Yes	360	6.6	965	10.9	2883	35.9	965	17
No	5102	93.4	7888	89.1	5148	64.1	4709	83
**Days of MPA**								
0	1262	23.1	1505	17	1108	13.8	1010	17.8
1–2	852	15.6	558	6.3	369	4.6	460	8.1
3–4	748	13.7	629	7.1	498	6.2	630	11.1
5–6	557	10.2	1062	12	450	5.6	755	13.3
7	2043	37.4	5099	57.6	5598	69.7	2820	49.7
**Days of VPA**								
0	2709	49.6	3824	43.2	3975	49.5	2582	45.5
1–2	754	13.8	735	8.3	771	9.6	545	9.6
3–4	606	11.1	646	7.3	899	11.2	499	8.8
5–6	410	7.5	1000	11.3	426	5.3	681	12
7	978	17.9	2647	29.9	1960	24.4	1367	24.1

Table 2 shows that the prevalence of good SRH was highest in Sri Lanka (73.7%) and lowest in Nepal (37.7%), while in Bangladesh over two-fifth (44.3%) and in India (58.7%) a little less than three-fifth of the population reported being in good health. Results of cross-tabulation also showed that people who reported good SRH were more likely to be in the younger age groups, female, currently married, having no formal education (except for Sri Lanka), self-employed, non-smoker, and never drinking alcohol. Those who reported engaging in any type of physical activities were also more likely to report being in good health.

**Table 2.** Self-rated Health (SRH) results breakdown in Bangladesh, India, Nepal and Sri Lanka, WHS 2002-03

	Bangladesh				India				Nepal				Sri Lanka			
	Good SRH (44.3)		Poor SRH (55.7)		Good SRH (58.7)		Poor SRH (41.3)		Good SRH (37.7)		Poor SRH (62.3)		Good SRH (73.7)		Poor SRH (26.3)	
	n	%	n	%	n	%	n	%	n	%	n	%	n	%	n	%
**Age**																
18–29	2092	38.3	1371	25.1	3444	38.9	1815	20.5	3100	38.6	1952	24.3	1838	32.4	607	10.7
30–39	1639	30	1393	25.5	2461	27.8	2010	22.7	2120	26.4	1831	22.8	1515	26.7	942	16.6
40–49	1005	18.4	1180	21.6	1478	16.7	1788	20.2	1446	18	1486	18.5	1265	22.3	1135	20
50–59	404	7.4	705	12.9	859	9.7	1328	15	747	9.3	1068	13.3	664	11.7	1265	22.3
60+	322	5.9	814	14.9	611	6.9	1921	21.7	626	7.8	1687	21	392	6.9	1725	30.4
P	<0.0001				<0.0001				<0.0001				<0.0001			
**Sex**																
Female	2797	51.2	3015	55.2	4241	47.9	4905	55.4	4578	57	4642	57.8	2865	50.5	3257	57.4
Male	2665	48.8	2447	44.8	4612	52.1	3948	44.6	3453	43	3389	42.2	2809	49.5	2417	42.6
P	0.002				<0.0001				0.175				<0.0001			
**Currently married**																
No	1191	21.8	1240	22.7	2125	24	1903	21.5	1333	16.6	1542	19.2	1765	31.1	1770	31.2
Yes	4271	78.2	4222	77.3	6728	76	6950	78.5	6698	83.4	6489	80.8	3909	68.9	3904	68.8
P	<0.0001				<0.0001				<0.0001				<0.0001			
**Educational attainment**																
Nil	2103	38.5	2354	43.1	2939	33.2	4046	45.7	4634	57.7	5116	63.7	227	4	579	10.2
Less than primaiy school	945	17.3	1043	19.1	691	7.8	1036	11.7	916	11.4	835	10.4	340	6	692	12.2
Primary complete	1431	26.2	1305	23.9	35	0.4	44	0.5	1205	15	956	11.9	1316	23.2	1748	30.8
Secondary complete	415	7.6	393	7.2	1470	16.6	1709	19.3	851	10.6	771	9.6	2326	41	1963	34.6
High school/equivalent	262	4.8	186	3.4	1328	15	876	9.9	297	3.7	201	2.5	1305	23	613	10.8
Pre-university/University	306	5.6	180	3.3	1142	12.9	531	6	137	1.7	145	1.8	159	2.8	74	1.3
P	<0.0001				<0.0001				<0.0001				<0.0001			
**Employment status**																
Govt. employee	235	4.3	169	3.1	425	4.8	204	2.3	257	3.2	233	2.9	550	9.7	397	7
Private employee	388	7.1	289	5.3	1107	12.5	779	8.8	177	2.2	161	2	721	12.7	437	7.7
Employer	2687	49.2	2021	37	4621	52.2	3444	38.9	5590	69.6	5051	62.9	2480	43.7	1923	33.9
Not working for payment	2157	39.5	2683	54.6	3594	40.6	4427	50	2008	25	2586	32.2	1918	33.8	2916	51.4
P	<0.0001				<0.0001				<0.0001				<0.0001			
**Smoking habit**																
Daily	2092	38.3	2261	41.4	2470	27.9	3222	36.4	2883	35.9	3116	38.8	704	12.4	783	13.8
Yes. not daily	262	4.8	257	4.7	257	2.9	274	3.1	418	5.2	482	6	443	7.8	494	8.7
Non-smoker	3108	56.9	2944	53.9	6126	69.2	5356	60.5	4730	58.9	4433	55.2	4528	79.8	4397	77.5
P	<0.0001				<0.0001				<0.0001				<0.0001			
**Ever drank alcohol**																
Yes	328	6	388	7.1	983	11.1	956	10.8	2819	35.1	2980	37.1	993	17.5	891	15.7
No	5134	94	5074	92.9	7870	88.9	7897	89.2	5212	64.9	5051	62.9	4681	82.5	4783	84.3
P	<0.0001				<0.0001				<0.0001				<0.0001			
**Days of MPA**																
0	863	15.8	2807	51.4	505	5.7	1726	19.5	353	4.4	4538	56.5	448	7.9	1566	27.6
1–2	1010	18.5	787	14.4	1346	15.2	637	7.2	883	11	715	8.9	811	14.3	488	8.6
3–4	770	14.1	590	10.8	593	6.7	673	7.6	498	6.2	795	9.9	635	11.2	596	10.5
5–6	590	10.8	410	7.5	992	11.2	1151	13	385	4.8	450	5.6	817	14.4	596	10.5
7	2228	40.8	874	16	5409	61.1	4666	52.7	5903	73.5	1534	19.1	2956	52.1	2428	42.8
P	<0.0001				<0.0001				<0.0001				<0.0001			
**Days of VPA**																
0	716	13.1	1469	26.9	735	8.3	4241	47.9	811	10.1	1478	18.4	601	10.6	3387	59.7
1–2	2584	47.3	847	15.5	3523	39.8	744	8.4	3638	45.3	402	5	2292	40.4	386	6.8
3–4	634	11.6	726	13.3	664	7.5	629	7.1	956	11.9	498	6.2	499	8.8	511	9
5–6	415	7.6	530	9.7	930	10.5	1098	12.4	418	5.2	562	7	755	13.3	482	8.5
7	1114	20.4	1890	34.6	3001	33.9	2142	24.2	2217	27.6	5084	63.3	1532	27	908	16
P	<0.0001				<0.0001				<0.0001				<0.0001			

Results of multivariable regression are shown in Table 3. Results indicate that Bangladeshis who engaged in 1-2, 3-4, 5-6, and 7 days a week were respectively 31% [AOR=1.306; 95%CI 1.085-1.572], 33% [AOR=1.326; 95%CI 1.093-1.609], 39% [AOR=1.389; 95%CI 1.125-1.716], and 46% [AOR=1.459; 95%CI 1.249-1.705] more likely to report being in good health compared to those who never engaged in MPA. In Sri Lanka, the odds of being in good health were respectively 49% [AOR=1.490; 95%CI 1.164-1.908], 80% [AOR=1.802; 95%CI=1.433-2.266], 2.3 times [AOR=2.255; 95%CI=1.805-2.817], and 86% [AOR=1.854; 95%CI=1.579-2.177] higher among those who those who engaged in MPA for 1-2, 3-4, 5-6 and 7 days a week.

**Table 3.** Multivariable analysis on the association between PA and SRH in selected South Asian countries, WHS 2002-03.

Variables	Bangladesh	India	Nepal	Sri Lanka
	Odds ratio (95%CI)	Odds ratio (95%CI)	Odds ratio (95%CI)	Odds ratio (95%CI)
**Days of MPA**				
0	-	-	-	-
1–2	1.306 (1.085–1.572)	0.913 (0.654–1.010)	1.186 (0.925–1.519)	1.490 (1.164–1.908)
3–4	1.326 (1.093–1.609)	0.926 (0.752–1.142)	1.289 (1.028–1.615)	1.802 (1.433–2.266)
5–6	1.389 (1.125–1.716)	0.894 (0.693–1.004)	0.930 (0.739–1.169)	2.255 (1.805–2.817)
7	1.459 (1.249–1.705)	1.055 (0.919–1.211)	1.478 (1.283–1.702)	1.854 (1.579–2.177)
**Days of VPA**				
0	-	-	-	-
1–2	0.854 (0.721–1.012)	1.024 (0.857–1.222)	1.110 (0.939–1.313)	1.995 (1.575–2.527)
3–4	1.025 (0.852–1.233)	1.103 (0.922–1.343)	1.178 (1.003–1.382)	1.255 (1.007–1.564)
5–6	0.962 (0.772–1.198)	0.959 (0.815–1.129)	0.894 (0.722–1.106)	2.036 (1.644–2.521)
7	1.212 (1.033–1.421)	1.340 (1.186–1.512)	1.363 (1.201–1.547)	2.224 (1.879–2.633)

In Bangladesh, India, and Nepal, those who engaged in VPA on daily basis were respectively 21% [AOR=1.212; 95%CI 1.033-1.421], 34% [AOR=1.340; 95%CI 1.186-1.512], 36% [AOR=1.363; 95%CI 1.201-1.547], 22% [AOR=2.224; 95%CI 1.879-2.633] more likely to report being in good SRH compared to those who never engaged in VPA. Among Sri Lankans, the odds of being in good SRH were respectively 2 times [AOR=1.995; 95%CI 1.575-2.527], 25% [AOR=1.255; 95%CI 1.007-1.564], 2.04 times [AOR=2.036; 95%CI=1.644-2.521], and 2.22 times [AOR=2.224; 95%CI 1.879-2.633] higher among those who engaged in VPA for 1-2, 3-4, 5-6, and 7 days a week.

## Discussion

This is one of the first studies that reports on the association between PA and SRH in a South Asian sample population. Findings of this study indicate a suboptimal level of PA among the adult population in South Asia. Within subgroups, variations were observed in PA. Findings showed that participants from Bangladesh had the lowest proportion of engaging in any type of PA. Similar results on low prevalence of PA were reported by previous studies from Bangladesh^33^ and India^34^. Findings suggest that the percentage of good SRH decreased with age in all countries. Female participants were more likely to report good SRH compared to/with males in all countries except for in India. A noticeable variation was observed in the prevalence of SRH among the four countries. Another important disparity was that having higher frequency of participation in PA did not always relate to higher rate of good SRH. For instance, compared to Sri Lanka, participants from Nepal had lower rates of reporting good SRH despite their higher frequency of involvement in both VPA and MPA. A possible connection might be higher living standards of Sri Lanka compared to the other countries measured in terms of Human Development Index (HDI). The correlation between higher educational status and better health outcomes are well documented across countries^18^^,^^19^. Our results further indicate that the rate of both moderate and vigorous type physical inactivity was highest in Bangladesh, followed by Sri Lanka, and India. Surprisingly, Nepal had lowest SRH despite its highest prevalence of MPA and second highest prevalence of VPA. As expected, engaging in regular PA was associated with higher odds of good SRH for most of the countries. In Bangladesh and Nepal, those who participated in MPA on a daily basis, and in Sri Lanka, those took MPA 5-6 days a week, had the highest odds of reporting good SRH. For VPA, highest odds of reporting good SRH were reported among those who exercised on daily basis, compared with those who exercised at a lesser frequency. Among all the countries, the strongest associations between SRH and PA in both categories was observed in Sri Lanka.

A major barrier to reporting association between SRH and PA is the lack of comparable studies reporting prevalence at the national level and the absence of standardised and validated instruments in studied countries^24^. Previous studies based on USA (86.2%)^20^ and Canada (89.9%)^21^ concluded that participants who rated their health as poor to average were less likely to take PA compared with those who rated their general health as good to excellent^21^. Similar findings were observed in South Korea, where an independent association between lower level of PA and poor SRH was reported^22^. Regarding the prevalence of PA, a study encompassing 76 countries reported that the prevalence of physical inactivity among individuals aged 15 years or older ranged from 3 to 62%^23^, which varied substantially from the worldwide prevalence of physical inactivity in adults of 31%^24^.

This study has some important limitations. Number of days of VPA and MPA (at least 10 minutes at a time) was used as a proxy for level of PA instead of exact duration. However, similar methodology was used in some other studies^21^^,^^26^. Another limitation is the absence of several necessary covariates which are commonly correlated with the level of PA, such as presence of disease conditions (diseased people are less likely to engage in PA), place of residency (urban and rural residences have differing patterns of engaging in PA), and other community level variables (e.g. neighbourhood cleanliness, safety, availability of public spaces for exercise). As the data were secondary, we had no control over the choice of selecting the covariates and the ways they were measured. For instance, we could not distinguish between work and leisure physical activity, which could have affected the associations. With the data being self-reported, there remains a possibility of under-and over-reporting, as well as the recall bias. Moreover, there are also differences in the way in which men and women describe their health. Women are more likely to report poorer functioning and worse overall health than men^27^. Last but not least, the results cannot be generalised to all age groups, since no data were available on participants below 18 years of age.

Further research is needed to better understand subgroup variations with larger sample sizes to address the heterogeneity found within South Asian groups in this study, who may have different motivations to undertaking and increasing their PA levels. At policy making level, attempts should be taken to encourage PA by improving the provision of necessary infrastructure and environment for exercise. This should be facilitated by developing national PA guidelines for people of different ages. Further studies should investigate the predictive factors of PA in the population and monitor the trends in PA to improve public health.
